# A Qualitative Study of the Experience of COVID-19 Patients in Burkina Faso

**DOI:** 10.4269/ajtmh.22-0351

**Published:** 2023-12-18

**Authors:** Blahima Konaté, Rachel Médah, Isidore Traoré, Samiratou Ouedraogo, Nongodo Firmin Kaboré, Ariane Kamga Mamguem, Oumar Billa, Dramane Kania, Hermann Badolo, Esperance Ouédraogo, Nathalie de Rekeneire, Armel Poda, Arnaud Eric Diendéré, Boukary Ouédraogo, Halidou Tinto, Tienhan Sandrine Dabakuyo-Yonli

**Affiliations:** ^1^ Institut des Sciences des Sociétés, Ouagadougou, Burkina Faso;; ^2^ Centre Muraz, Bobo-Dioulasso, Burkina Faso;; ^3^ Université Nazi Boni, Bobo-Dioulasso, Burkina Faso;; ^4^ McGill University, Montréal, Canada;; ^5^ Epidemiology and Quality of Life Research Unit, INSERM U1231, Georges François Leclerc Comprehensive Cancer Centre, Dijon, France;; ^6^ Observatoire National de Santé Publique, Ouagadougou, Burkina Faso;; ^7^ Institut de Recherche en Sciences de la Santé, Ouagadougou, Burkina Faso;; ^8^ Institut Pasteur du Cambodge, Phnom Penh, Cambodia;; ^9^ Centre Hospitalier Universitaires Sanou Souro, Bobo-Dioulasso, Burkina Faso;; ^10^ Centre Hospitalier Universitaires Bogodogo, Ouagadougou, Burkina Faso;; ^11^ Direction des Systèmes d’Information en Santé, Ouagadougou, Burkina Faso;; ^12^ Institut de Recherche en Sciences de la Santé, Nanoro, Burkina Faso

## Abstract

In Burkina Faso, the health system is characterized by systemic insufficient and antiquated health-care infrastructures. Consequently, few health-care establishments have the required resources to diagnose and manage patients with COVID-19, and fewer still have intensive care facilities for severely ill patients with COVID. Furthermore, there is a widespread scarcity of qualified health-care staff. The aim of this study was to explore the experiences of patients with COVID-19 who recovered after being cared for in Bobo Dioulasso and Ouagadougou. Using individual semistructured interviews, we performed a cross-sectional qualitative, descriptive study from June 12 to 30, 2020 with the aid of 13 well-educated patients who had survived COVID-19. The results reveal that prior to hospital admission, the main reason that prompted patients to seek care was onset of symptoms of COVID-19, regardless of whether they had been in contact with suspected or confirmed cases. Transmission was mainly believed to have occurred in the community, in the hospital, and during travel. Patient management was punctuated by frequent self-medication with medicinal plants or pharmaceutical drugs. The participants reported a negative perception of hospitalization or home-based management, with several forms of stigmatization, but a positive perception influenced by the satisfactory quality of management in health-care centers. This report of patient experiences could be helpful in improving the management of COVID-19 in Burkina Faso, both in the health-care setting and in home-based care.

## INTRODUCTION

The SARS-CoV-2 virus and its resultant disease, COVID-19, first emerged in Wuhan, China, in late December 2019.^1^^–^^3^ By January 30, 2020, the spread of the disease was such that the WHO declared COVID-19 to be a public health emergency on a global scale. Since then, the number of cases and deaths related to COVID-19 has continued to increase, with waves of infection of varying intensity. With the advent of vaccines against COVID-19 in 2021, there was a drop in severe cases resulting in death and, to a lesser extent, a reduction in incidence,^4^ but incidence was on the rise again in December 2021 with the appearance of the omicron variant.^5^ The Americas, Europe, Southeast Asia, and the Mediterranean region have been hardest hit, together accounting for ∼94% of confirmed cases and deaths as of November 24, 2021. Compared with these regions, Africa has reported lower numbers of COVID-19 cases. However, from March 2020 to April 2022, WHO data showed an increase in the number of cases reported in Africa: 3,312,273 confirmed cases and 82,741 deaths.^6^

Recent studies have shown that COVID-19 morbidity and mortality in Africa are much higher than previously thought. According to one study,^7^ the excess deaths resulting from SARS-CoV-2 (direct and indirect deaths) in Burkina Faso is 67.18 times greater than the official figure.^7^ Another study^8^ by the WHO reported that up to 65% of Africans have been infected by SARS-CoV-2—a figure that is 97 times greater than the number of confirmed cases reported officially.

Burkina Faso is a landlocked Sudano-Sahelian country situated in the heart of West Africa. The population is estimated at 20,478,979 inhabitants, and 64.2% of the population is younger than 24 years, 77.9% is younger than 35 years, and 3.4% is 65 years or older.^9^ The most populous cities are Ouagadougou and Bobo-Dioulasso, and together account for 62% of the entire urban population.^9^ In terms of health care, Burkina Faso is organized in 13 sanitary regions and 70 sanitary districts, with six university teaching hospitals; a national surveillance system for severe, acute respiratory infections; and a national influenza laboratory.^10^ In Burkina Faso, the first case of COVID-19 was diagnosed in March 9, 2020. As of November 14, 2021, 15,514 confirmed cases and 265 deaths had been reported,^4^ with the majority of cases observed in the main cities—namely, Ouagadougou and Bobo-Dioulasso. To respond to the pandemic, a national emergency response plan was elaborated with national directives for the management of COVID-19 cases in early 2020 by the Ministry for Health. National directives were introduced in February 2020 for the management of COVID-19 cases, which specified the case definition (suspected cases, contact cases, and confirmed cases), and recommended management and preventive measures to be implemented in the health-care and community settings. The directives also summarized the clinical aspects of the disease, according to available knowledge at the time, such as the duration of incubation, and signs and symptoms, as well as the biological diagnosis.^11^ Several treatment centers for confirmed cases were opened throughout the various regions of the country.^12^ However, in Burkina Faso, as in the majority of African countries, health-care infrastructures are outdated and in poor condition.^13^^,^^14^ As a result, existing hospitals are incapable of managing all the patients presenting with COVID-19. Indeed, few hospitals have the means to diagnose and manage patients, and fewer still have intensive care facilities available to manage critically ill patients with COVID-19. This situation is compounded further by the lack of qualified staff.^15^^,^^16^ These conditions could affect the management of patients negatively, despite the implementation of specific measures to bolster health services, including isolation of confirmed cases in their home, providing extra supplies to hospitals, and training extra staff. In this context, feedback from patients who survived COVID-19 would be helpful in improving management. The objective of this study, therefore, was to explore the experience of patients who recovered from COVID-19 after management in Bobo-Dioulasso and Ouagadougou, which can only be accessed by eliciting a direct narrative through interviews. Qualitative inquiry is thus the methodology most suited to this purpose, because its aim is to collect first-hand reports of qualitative, lived experiences by asking the people who have had the experience in question to recount the phenomenon in their own terms.

## MATERIALS AND METHODS

We performed a cross-sectional qualitative, noncomparative study from June 12 to 30, 2020 in Bobo-Dioulasso and Ouagadougou, the two main cities of Burkina Faso. We recruited patients who had recovered from COVID-19 after having been managed by reference health-care centers in Ouagadougou (the Bogodogo University Hospital) and Bobo-Dioulasso (Pediatric Hospital of Belle Ville). Contact with the patients was established through the intermediary of the competent authorities in each hospital, who had previously obtained the oral consent of the patients to be contacted about participating in an interview. Hospital staff members were not aware of the content of the interview guide (described later) or the subject of the interview (notably, questions about how the patient’s hospital stay was perceived). Interviews were performed in person or by telephone by four researchers (two teams of two researchers, one team per site). All interviews were conducted in French or in the local languages—namely, Mooré (in Ouagadougou) or Dioula (in Bobo-Dioulasso). Subjects were recruited on the basis of volunteer participation. Only patients who were eligible and accepted to be interviewed could be included, which may have limited the diversity initially intended in the study sample.

The interviews followed the interview guide developed in advance by a team of qualitative researchers prior to this study, based on a review of the literature and personal experience of the concerns of the Burkinabe population. The interview guide covered two main themes: the patients’ experience prior to hospitalization (covering points such as screening, diagnosis, sharing of the positive diagnosis with family/friends, and therapy) and their experience during hospitalization (covering points such as treatment, food, follow-up, relations with the caregiving team, and hygiene measures). The interview guide is provided as Supplemental Appendix 1. Interviews were audio-recorded and fully transcribed in French, and were analyzed using thematic analysis. The main steps of thematic analysis were implemented as described by Albarello^17^ and Braun and Clarke.^18^ First, we proceeded with an in-depth reading of all the discourse, with open coding. The first round of open coding was performed by two researchers independently (K. B. and M. R.). Then, codes were regrouped to identify categories with relevant subthemes. Meetings were held with the full research team (all authors) to reach a consensus on the main themes to be retained and the corresponding subthemes. Any conflicting interpretations were resolved by discussion within the group until a consensus was reached. Themes, subthemes, and quotations were translated after the analysis by a professional medical writer with a scientific PhD, who is also an experienced qualitative researcher. QDA Miner 4.0 software was used to assist with data management and analysis. With the consent of the participants, illustrative quotes from the interviews are shared in support of our results.

## RESULTS

### Study participants.

Thirteen patients were interviewed (six in Ouagadougou and seven in Bobo-Dioulasso), including six men and seven women. Age ranged from 20 to 80 years. The majority of the participants were married and had a secondary school–level education. They had all contracted COVID-19 between October 2020 and January 2021. Nine persons were isolated in a hospital and four stayed at home during their isolation period. The majority of study participants included teachers (Table 1).

**Table 1:** Characteristics of the study population

Characteristic	*n* (%)
Sex	
Male	5 (38.5)
Female	8 (61.5)
Level of education	
None	3 (23.1)
Primary	0 (0)
Secondary school	3 (23.1)
Third level	7 (53.8)
Place isolated	
Hospital	9 (69.2)
Home	4 (30.8)
City	
Bobo-Dioulasso	7 (53.8)
Ouagadougou	6 (46.2)
Age, years	
20–59	9 (69.2)
≥ 60	4 (30.8)
Profession	
Housewife	2 (15.4)
Teacher	5 (38.5)
Student	2 (15.4)
Other	4 (30.8)

The two main themes to emerge from the discourse are described in the following sections: 1) the patients’ experience prior to hospitalization, including their experience of being tested and diagnosed; sharing the information about their COVID-positive status with their entourage; the reactions of their friends and families; and their therapeutic journey; and 2) the patients’ experience of hospitalization, including their treatment, food, follow-up, experience of the hospital setting, interactions with caregivers, and local hygiene measures.

### Reasons prompting the patient to get tested.

The participants in our study cited several reasons that prompted them to get tested. First among them was the onset of symptoms suggestive of COVID-19, as indicated by one patient:

In the meantime, I wasn’t feeling well, and I thought I had malaria. We started to treat for that at home, but then I realized that it was more complicated than that, so I went to the Saint Camille hospital and the doctor there noticed that I was coughing a lot, and that’s when he advised me to get a COVID test. I did it, and a second test confirmed the first one; I was positive for COVID-19. (Patient 2, male, Ouagadougou)

The patients believed their symptoms occurred predominantly after contact with a suspected or confirmed case in the community, as illustrated by this patient health-care worker:

It all started on 16 December 2020. I started feeling the signs. I was coughing a lot, [it] was getting . . . hard to breathe, I had a pain in my chest. In my department, there was a suspected patient. And since I was on duty, I was in contact with him a lot. I did have a mask. It was only a suspected case, because he didn’t have the results of his test yet. I had planned to do his test, so when the team came to take the sample, I got them to take a sample from me too, since I was a contact case. They took a sample and the next day they rang me to say that both myself and my patient were positive. (Patient 2, female, Bobo-Dioulasso)

Although this transmission was thought to have occurred in the hospital setting, other participants believed they had probably been infected in their family circle, as explained by this patient:

Last December, I was sick. and with the pains and symptoms of COVID that I saw on the TV, I began to think that maybe I had COVID. I tried to search for more information on the Internet and I found that the symptoms were common in my situation. The next day, my daughter, who was supposed to be going away to study, got her test back and it was positive, so I understood that one of us had contaminated the other. (Patient 4, male, Ouagadougou)

Another patient declared having contracted COVID at a party:

Yes, I got COVID at a party. There were at least 100, if not 200, people there. And it was after the party that I started to feel bad—fever, headache . . . . So you have to avoid large gatherings as much as possible. (Patient 1, female, Bobo-Dioulasso)

Yet another patient declared having contracted COVID while traveling abroad:

Well, the reasons why I went to get test are these: I had a mission to do in Bamako, and 2 weeks after coming home, I started feeling signs of COVID-19—headache and other things. And also I was in contact with a Belgian colleague when I was in Bamako, and when he got home, he tested positive for COVID-19. So between that and all the signs, I thought, *Well I better go and do the test*, even though I wasn’t certain. (Patient 3, female, Ouagadougou)

Other participants who had had no symptoms had gone for testing after being in contact with a COVID-positive person:

About the test, we had a colleague, our project coordinator, who was supposed to return to her own country. She went for a COVID test and it was positive. That’s when they called us in the middle of the night, around 1 or 2 o’clock in the morning, to advise us to get tested. So I went to Pogbi hospital to do the test and they told us there that we wouldn’t have the results for 72 hours. I was positive. (Patient 5, female, Ouagadougou)

Last, some participants declared having tested positive for COVID-19 after being hospitalized for another disease, the symptoms of which were similar to those of COVID-19, as explained by this patient:

It started with headaches and we went to the clinic. They said it was malaria or dengue and I was in hospital there for 5 days with an infusion. Then on the Monday, they let us out and we came home. Monday, Tuesday, and Wednesday, the breathing difficulties started. Someone told us there was a pulmonologist at the St. Leopold clinic, so we went there. (Patient 2, female, Bobo-Dioulasso)

### Perception of COVID-19 testing.

The analysis of the participants’ perceptions of COVID testing was related to having samples taken, and the announcement and sharing of results. Indeed, contrary to HIV or other viruses, COVID-19 testing is done by nasopharyngeal swab. For most patients, this procedure is most unpleasant, as illustrated by the following patient:

During the sample, it felt unpleasant. I’m old, after all, but I wouldn’t say it hurts. It’s just unpleasant. (Patient 2, male, Ouagadougou)

Apart from the unpleasant sensation, other patients mentioned a feeling of suffocation, difficulty breathing, or having a cold when the sample was taken. In addition to the actual sensation of the test, some patients complained about the delays, the poor organization of some test centers because they ran out of reactants, or the lack of logistic material or human resources, as recounted by the following patient:

When we arrived, the team that was there had to leave, and another team was supposed to come, and that all took more time. Then when they wanted to start, they tried to do the interview with everyone first, then take the samples. So I went in to complain. I said, “What is this organization about? You can’t do the interview with everyone first before doing the sample.” The guy who was there said, “Ma’am, I’m on my own and I can’t do everything.” So I can tell you, that day, I didn’t appreciate the work organization. (Patent 3, female, Ouagadougou)

The long wait time for having a sample taken was mentioned by several participants:

We didn’t have too much difficulty for the test, except that we were supposed to do the test Friday, but there were too many people. So instead, I went on Saturday and I was ninth on the list. They need to increase the number of tests so that people don’t have to wait so long. I could’ve died from it before I had a chance to do the test. (Patient 6, male, Ouagadougou)

After the sample has been taken, the results have to be returned to the patients, and the experience of the announcement of a diagnosis was different across participants. For some, they received a text message on their cell phone or by a social network (such as WhatsApp), or a phone call, either directly or to someone else, to inform them that their test result was positive. For others, particularly participants who had been hospitalized, they were given their diagnosis in person by a health-care professional. Although most participants did not have any issues with the various forms of communicating the diagnosis, some were unhappy with the manner in which they were told of their COVID positivity, for example:

It was a nurse who did it. He came out and stood in the middle of the room and started shouting. If he had been young, I might’ve understood, but it was an adult who did that. I didn’t like it. It wasn’t right to come and announce the result in front of the person like that. He should have called the person aside, or at least asked if there was a father or a brother he could give the result to. But to call it out like that in front of the patient, that wasn’t nice. (Patient 3, female, Bobo**-**Dioulasso)

Mentioning or announcing the results provoked different reactions across the participants. Some participants admitted that they were shocked, primarily because they had no signs or symptoms of COVID-19:

The result was a shock to me. I was surprised because I had no signs, no symptoms of the disease. So I couldn’t understand why I had got the virus. (Patient 1, female, Ouagadougou)

The result was also important for some participants whose health state put them at risk of developing a severe form—in particular, those with diabetes or respiratory disease. Such was the case for the following patient, who expressed worry about her positive diagnosis, preventing her from sleeping:

Yes, getting the results was a shock to me. I wasn’t expecting to be positive, and since I have allergies and asthma, and since I keep hearing that asthmatics and diabetics are vulnerable, well, I felt vulnerable. I had difficulty driving from the bank where I was when they gave me the result, back to the medical center of Pissy. And then that night, I really didn’t sleep well. (Patient 3, female, Ouagadougou)

### Sharing the results and the reactions with the participants’ entourage.

The majority of participants reported that they told their close relatives about their COVID-19 diagnosis (spouse, uncles, aunts, siblings, children, etc.):

I told the news to my parents first, because I don’t live with them in Ouagadougou. Honestly, when I heard, I called my two brothers in Ouaga, I told them I was in the district of Bogodogo, that I finally did the test, and that it was positive. (Patient 1, female, Ouagadougou)

In addition to close relatives, some participants said they informed their boss, director, work colleagues, or contact persons. The participants reported that this was in the aim of allowing these contact persons to get tested and stop the transmission of the virus. In both the family and work circles, the reaction of the participants’ entourage was generally judged to be quite positive for the majority, as indicated by the following patient:

Yes, yes. People were calling me to provide support. In any case, I had good psychological support from my family and work colleagues. (Patient 3, female, Ouagadougou).

However, some participants reported they were the victim of several forms of stigmatization on the part of their entourage. For example:

At 12 o’clock, I was at the water tap, and it was the time when the children are let out. There was a lady from the crèche sitting over to the side, not far from the tap, and when I started to approach her, she started saying, Heeeey, don’t come near me. I don’t want that COVID-19 to catch me. (Patient 5, female, Ouagadougou)

Another patient also reported having felt stigmatized:

Of course, there were people who were suspicious. They were talking about it until a doctor actually dared to say no, that apparently teachers had come and been contaminated by me. Well, there was stigmatization and suspicion from certain people toward me. Definitely, the stigmatization was there. (Patient 1, female, Bobo-Dioulasso)

Other concerns about stigmatization were also reported, such as colleagues failing to greet someone because they had been to visit a person sick with COVID-19, or refusing to participate in the funeral of a person who died of COVID-19.

### The care pathway.

Before the initiation of treatment, participants reported they followed divergent care pathways. A first group of participants (a minority) reported their only contact was with a health-care center, as illustrated by the following patient:

When I got back from the city, I felt sluggish. There’s a clinic nearby, so I went there, but they said it must be malaria, and they gave me medications that I took. But, 2 days later, I still didn’t feel better. I had problems. I was coughing. My children brought me to a medical center with a surgical unit: The Source. When he heard all that, the doctor there decided to do the test. (Patient 6, male, Ouagadougou*)*

With the exception of a few patients, most participants acknowledged that their first reaction was to self-medicate, using modern drugs (mostly cough remedies, antipyretics, antimalarial drugs, and vitamins). In addition, some participants also reported using plant-based medicines:

I tried to boost myself. In the end, I went and scraped the bark off those trees for the cough, and there were other things. It’s grains that I gathered from trees. I was told that they can cure a cough, so I used those, after all. (Patient 1, female, Bobo-Dioulasso)

When these treatments failed, the second option was to use public or private health-care structures, as indicated by this patient:

When I was in pain, I took paracetamol. I took them until the whole packet was gone. When I take this drug, it gets better and I sleep. But the next day, it hurts again. My head was really painful. It hurt most at night. And one night, it got worse. So that night, we went to the clinic. (Patient 2, female, Bobo-Dioulasso)

### Participants’ experience during hospitalization.

Management of the patients with COVID-19 started after the sampling, confirmation, and announcement of the positive result. Asymptomatic patients or those with only mild disease were isolated and monitored at home, whereas those with severe symptoms were hospitalized and managed in accordance with a strict protocol. The participants’ perception of their management related to several aspects—namely, treatment, food, follow-up, relations between caregivers and the patients, and the hygiene measures in the hospital.

In this regard, several participants reported they did not appreciate that the hospital staff obliged them to take medications very early in the morning, even before they had had time to wash or get dressed, as reported by one patient:

Very early in the morning, they made me take the medication, even though I hadn’t even had time to get washed. I complained about it, but the next day, they did exactly the same thing again. (Patient 1, female, Bobo-Dioulasso)

Other patients complained about having to pay for the medication, even though the authorities had said that patients with COVID-19 would be treated for free. One patient recounts his experience:

No the treatment wasn’t free. I don’t know if it was subsidized, but it wasn’t free of charge. I paid for everything. Yet they had said that it would be free. To start with, I had to pay 35,000 FCFA [53 €] for a kit each for my wife and me. I paid everything from start to finish. I think I might have spent almost 200,000 francs [305 €] with the whole thing. I remember, there was an injection that they were giving us twice a day, and it cost 7,500 Franc de la Communauté financière africaine (FCFA) [11 €] per injection. And everyone in the ward paid. The tests, we paid for all those. I defy anyone who says it’s free. I have all the prescriptions and receipts. This thing about it being free in Burkina Faso, I don’t believe that. (Patient 4, male, Ouagadougou)

In addition, some participants had criticisms to level at the organization of the meal service for hospitalized patients. First, there were delays in the distribution as a result of a delay in putting the meal platters together. Lunch was served at 2 pm. Then, there was no breakfast for patients who had no accompanying person to provide it for them, as noted by this patient:

There’s one thing I would like to say, and it’s a problem for patients who have no one accompanying them. It’s that there is no breakfast for the patient. If you don’t have someone accompanying you, then you get no breakfast. I had my son who brought me my porridge every morning. But the others, they had to wait until lunchtime to get something to eat. But when you’re sick, that’s not easy. That’s what I saw in the way of difficulties. (Patient 6, male, Ouagadougou)

Several other participants, especially those who were in isolation in their home, reported that the schedule for testing during follow-up was not respected. In this regard, one patient said he waited 3 weeks to have a confirmatory test, despite several phone calls to the team who were supposed to take the sample. Furthermore, some patients found the home isolation difficult, and felt like they were in prison:

The difficulty was not that I couldn’t have contact with my family. My granddaughter, who is usually with me all the time, I couldn’t touch her, and I really missed that, and I couldn’t share the living room with everyone. I was always put aside. You feel like a prisoner . . . . It’s a prison that doesn’t say its name. (Patient 3, female, Ouagadougou)

Concerning the relationship between caregivers and patients, few respondents had any criticisms to level at the health-care staff involved in their management. Only a few patients had minor complaints about the behavior of the staff, such as the following patient who complained about a nurse:

The day I arrived there, I was met by a nurse who started scolding me. She asked me if I had diabetes or blood pressure, questions like that. The nurse was talking to me as if we were in conflict. (Patient 3, female, Bobo-Dioulasso)

Some other respondents reproached the staff with not respecting the follow-up procedures they themselves had given to the patients. However, despite these criticisms, the majority of respondents had a positive view of their management in the health-care centers. The warm welcome afforded by the staff was greatly appreciated, as shown by the following quotes:

Honestly, at the welcome desk, they were respectful. There was a good atmosphere. When I arrived, I was set up in a room, and even when the teams changed over, it was the same good atmosphere. (Patient 2, male, Ouagadougou)

We were met by a doctor called X who welcomed us really warmly. I really want to underline that. It made me feel confident. (Patient 4, male, Bobo-Dioulasso)

In addition to the warm welcome, several patients emphasized the availability of the staff, as cited by the following patients:

I have to say that the medical staff really took good care of me and my wife, because we were both sick. They took really good care of us. And I could see that they did the same for all the other patients—in the regularity of care, the courtesy in the way they spoke, and, on top of that, professionalism, to raise our spirits. (Patient 4, male, Ouagadougou)

They were always available for us. Every half hour they came by to see us, and there was even a camera. If you needed something, you could just raise your hand and someone would come to see what you needed. (Patient 2, male, Ouagadougou)

Participants confirmed unanimously that all staff members respected hygienic measures scrupulously in both cities:

Everything was clean. They cleaned the rooms three times a day. They removed all the trays with our meals on them. Immediately, as soon as you were finished eating, they came around and took everything away, and there was no joking about it. In terms of hygiene, it was impeccable. (Patient 4, male, Bobo-Dioulasso)

In addition to these measures, some respondents stated they had a positive perception of the reception they received, and the punctuality, availability, courtesy, and communication of the staff, as stated by the following patient:

They were available, of course, as soon as I called, they came. There was no problem, they took very good care of me (Patient 2, male, Bobo-Dioulasso)

## DISCUSSION

This study aimed to explore the experience of patients with COVID-19 in Burkina Faso, from the discovery of their diagnosis, through to the end of hospitalization. The participants’ discourse reveals that the majority were prompted to get tested because they were symptomatic. Some participants reported feeling somewhat stigmatized by their status as COVID positive, despite being predominantly well supported by their family and entourage. Overall, the participants reported a globally positive experience with hospitalization, particularly the warm welcome afforded by the staff and the scrupulous hygiene in the COVID treatment centers. The results of our study show that the main reason participants got tested for COVID-19 was because they started to have symptoms, regardless of whether they had been in contact with a suspected case or contact case. The main symptoms cited were fever (as with malaria), cough, breathing difficulties, chest pain, and headache.

Several studies^19^^–^^22^ performed in Africa among the general population, health-care workers, or even patients with COVID-19 report similar findings. The circumstances of diagnosis were principally a result of the presence of symptoms in the population interviewed in this study. This confirms the WHO findings that diagnosis of COVID-19 in Africa was mainly among symptomatic cases presenting to health-care providers, thus leading to undernotification of cases. Again according to the WHO, only 14.2% of all COVID-19 infections in Africa were detected, which corresponds to approximately one infected person in seven.^23^

Analysis of the discourse of our respondents reveals that the participants believed there were three main modes of transmission. The first mode was community transmission—within the family circle or at gatherings. This may be explained by suboptimal implementation of social distancing, principally as a result of community cultures that characterize our society. Indeed, participation in funerals, weddings, baptisms, and visits during traditional religious feasts is considered mandatory by many Burkinabe, even though they see these events as potential sources of infection.

Second, some believed they had nosocomial transmission in the hospital. This is in line with the particular vulnerability of health-care staff to COVID-19 during the pandemic. Indeed, they incurred a high risk not only of getting infected from their patients, but also of spreading the infection unknowingly, especially if they failed to take adequate precautionary measures.^24^ Since July 2020, the WHO observed that more than 10,000 health-care workers in Africa were infected by COVID-19, accounting for more than 5% of all infections in 14 countries of sub-Saharan Africa alone. In four of these countries, infections among health-care workers accounted for more than 10% of all infections.

The third suspected source of infection was via travel. The fact that the virus can enter the country with travelers was the underlying justification for border closures in many countries, and continues to underpin the reinforced hygiene and security measures at border crossings. However, it should be noted that there is no way participants could know with certainty how or when they were in contact with the virus, and there is undoubtedly a discrepancy between their beliefs and reality. In Africa, the majority of SARS-CoV-2 transmissions were via asymptomatic individuals. The WHO estimates that 80% of SARS-CoV-2 infections in sub-Saharan Africa were asymptomatic.^25^

The patients interviewed in this study reported on their management, in-hospital for some and at home for others. This is line with the national guidance for COVID-19 management in Burkina Faso, which stated that patients with suspected or confirmed cases of COVID with signs of severity should be admitted to the hospital for management in dedicated, isolated units, using standard precautions, with additional precautions for close contacts. Cases without signs of severity could be management at the site of diagnosis, or at home, taking all the necessary precautions.^11^

Our respondents reported having experienced stigmatization, such as verbal attacks, physical distancing seen as rejection, or refusal to attend the funeral of a person who died of COVID-19. Similar phenomena have been reported in the literature regarding these forms of stigmatization. For example, stigmatizing has been reported in Egypt^26^ and India.^27^ This form of reaction could be interpreted as the stigma of labeling,^28^^,^^29^ insofar as the infected persons are labeled as being “different” or to be feared, because of their state of health. Other patients reported they had the full support of their entourage, including neighbors, families and colleagues. According to the WHO, family support remains a key form of support to COVID-positive individuals who are hospitalized.^30^ A study by Sun et al.^1^ of 16 COVID-positive patients who were hospitalized reported that the support and encouragement of family, neighbors, and friends was perceived very positively during hospitalization. The study respondents faced difficulties to discern proven protective measures (physical, distancing and avoiding crowed and poorly ventilate spaces etc.) from non-proven measure that may be stigmatizing (mandatory quarantine with inadequate support limiting access to healthcare facilities or professionals. This suggests that stigmatization derived from a misunderstanding of the epidemiology of COVID-19. This has actually been reported by others^31^^–^^33^ in the context of other pandemics such as HIV/AIDS. Furthermore, these perceptions mirror the ambiguity of the concept of stigmatization insofar as it is difficult to distinguish the behaviors that are purely stigmatizing, from behaviors that are intended to prevent the spread of COVID-19, and the same behavior may be interpreted as stigmatizing by some, but protective by others.

The patients in our study had an almost universally positive appreciation of the quality of the management they received in the dedicated COVID-19 centers, especially the reception, communication between caregivers and patients, the availability of the health-care staff, and cleanliness. One study, performed in Iran,^34^ highlighted the importance of the kind words and empathic attitude of nurses and other health-care staff in charge of managing patients with COVID**-**19. In a study from the United Kingdom,^35^ COVID-19 patients reported a positive experience of the quality of care received, which they rated at 9 of 10. In Nigeria, a study^22^ among 11 patients with COVID**-**19 found that the participants were impressed by the hygiene of the health-care centers and the quality of care. Yet, it has been reported in the literature that we live in a context of “inhospitable medicine,”^36^ where patients generally perceive those delivering health care to be hostile in developing countries.^37^ The abrupt change in this attitude could be explained by the large volume of resources that was invested by the Ministry for Health and its partners to fight against COVID-19 in Burkina Faso. Indeed, as soon as the first patient with COVID-19 was notified in the country on March 9, 2020, the government, together with its financial and technical partners, and economic and other stakeholders, came together to finance training for health-care personnel and to provide financial incentives to equip health-care centers with supplies and equipment—notably, personal protection equipment, and prevention and disinfectant supplies.

The analysis of the care pathway followed by the patients in our study shows the high rate of use of self-medication with medicinal plants or conventional drugs to treat cough, malaria, or pneumonia. In Togo, Sadio et al.^38^ reported that the overall prevalence of self-medication to prevent COVID-19 was 34.2%, and that the most widely used products were vitamin C, traditional medicine, chloroquine, or hydroxychloroquine. This practice can most likely be explained by prevailing false ideas that traditional African medicine can cure or prevent all diseases,^39^ or the idea that chloroquine can treat COVID-19. It may also be the result of family habits or contextual factors, as suggested previously by Schmitz.^40^ The use of traditional medicine is common in Burkina Faso, and was estimated at 85% in one recent study.^41^ In Ethiopia, Umeta Chali et al.^42^ reported that 46% of respondents used traditional medicines for the prevention and/or treatment of COVID-19, and they listed the 32 most frequently used plant-based medicines, which included garlic, ginger, and lemon.

Our study has some limitations. First, the participants all came from two major COVID-19 care centers—namely, the University Hospital Bogodogo in Ouagadougou and the University Hospital in Bobo-Dioulasso. However, these are the main two isolation centers for COVID-19 in the country. Second, a major limitation of this study is the social composition of the panel. Indeed, the lack of diversity may have precluded taking into account the experiences of other social groups, which might have enriched our findings. Indeed, only patients who were eligible and accepted to be interviewed could be included, which may have limited the diversity initially intended for the study sample. Next, the cross-sectional nature of our study precludes any insights into changes in management that may have occurred during the course of the epidemic, thereby limiting the generalizability of the results. To account for this, we had planned to include patients from very early in the management up to very recently; but, faced with a lack of medical data in the hospital databases, particularly for patients early in the epidemic, we could only include patients who were treated between October 2020 and January 2021.

This study raises some interesting perspectives to inform future management. Although our study focused on two cities, with limited diversity in the sample, it nonetheless provides interesting insights and detailed experiences of screening and treatment, and the experience of hospitalization. This is important in view of the paucity of literature on this point. In general, the persons interviewed in our study had a positive overall experience of their management. However, they raised some complaints regarding their interactions with the health-care environment—notably the fact that the care was not free of charge, although the government had promised it would be. Taking these issues into account, and ensuring equal access to care at no charge in particular, could help to improve management of this disease in the future.

## CONCLUSION

The results of this study show that patients diagnosed with COVID-19 in two major cities in Burkina Faso had diverging experiences with regard to their management, although the majority of patients reported a positive experience during their hospital stay. Differences related to the motives that prompted them to get tested, their care pathways, and the interactions they had with the caregiving staff. The patients’ experiences reported here could provide pertinent insights that could be used to inform future management and care, especially in settings with limited resources, where international guidelines may not be applicable.

## Supplemental files

10.4269/ajtmh.22-0351 Supplemental Materials
