# Supplementary material for: A Qualitative Study of the Experience of COVID-19 Patients in Burkina Faso
Source: Am J Trop Med Hyg. 2023 Dec 18;110(1):170–8. doi: 10.4269/ajtmh.22-0351 (PMC10793024; doi:10.4269/ajtmh.22-0351)
Supplement: Supplemental Materials [file tpmd220351.SD1.pdf]

Étude multidisciplinaire de recherche sur la maladie COVID-19 au Burkina Faso (EMuL-COVID-19), ANRS-COV13 : volet socio-anthropologique

Guide d'entretien pour appréhender le vécu des patients pris en charge dans les structures de santé

I. Introduction

Bonjour/Bonsoir. Je m'appelle ________________________Nous sommes des agents envoyés par le Centre MURAZ pour une étude sur le coronavirus (COVID-19). Cette étude a pour objectif principal d'analyser le parcours thérapeutique et le vécu des patients pris en charge dans les centres dédiés aux personnes infectées par le SARS-CoV-2 ainsi que les perceptions, l'acceptabilité et l'application des stratégies de prévention de l'infection par le personnel soignant et la population générale. Les informations que vous accepteriez nous confier seront confidentielles. L'entretien prendra environ **45 minutes**. Avec votre permission, nous allons utiliser un enregistreur pendant l'entretien afin de retranscrire fidèlement vos propos par la suite. Nous vous remercions d'avance pour votre participation

II. Discussions approfondies

Vécu de la maladie chez le patient de la COVID-19

1. **Vécu de la maladie avant l'hospitalisation**

- Dépistage et raisons du test : Qu'est-ce qui vous a amené à faire le test ?
- Prélèvement : Pouvez-vous nous parler du prélèvement (quand ? appréciation, difficultés rencontrées)
- Annonce des résultats : Quelles appréciations faites-vous de l'annonce des résultats (durée, choc que cela a causé, appui psychologique ou non)
- Partage du résultat et réaction de l'entourage : Avec qui avez-vous partagé (soutien, rejet, stigmatisation etc.)
- Confinement à domicile : Quelles sont les mesures barrières à respecter, difficultés rencontrées etc.)
- Traitements suivis avant la mise sous traitement : quels sont les traitements suivis avant la mise sous traitement (automédication, médicament traditionnel, moderne, recours tradipraticiens, personnel médical, groupe organisateur de soins)
- Perception de la COVID-19 pendant cette période : Comment percevez-vous la COVID-19 ?
- Changements dans votre vie : quels sont les changements (positifs, négatifs) dans votre vie ?

- Suggestions pour un meilleur dépistage

2. **Vécu de la maladie pendant l’hospitalisation**
    - Traitements reçus : Pouvez-vous nous parler des traitements reçus dans le centre de santé ? (Type de traitement : comprimé, perfusion, prélèvements effets secondaires, appui psychologique reçus)
    - Autres traitements suivis pendant et après la prise en charge : quels sont les traitements autres que ceux préconisez par le centre de santé que vous avez fait pendant et après votre hospitalisation ?
    - Relation soignant/patient ? Pouvez-vous nous parler de vos relations avec les différents personnels de santé dans la chaîne de prise en charge (accueil, disponibilité, communication, appréciation du comportement : raconter des expériences vécus)
    - Organisation de la prise en charge : quelle appréciation faites-vous de l’organisation de la prise en charge dans la formation sanitaire (hygiène des lieux, exiguïté des espaces, alimentation, isolement des patients, heures d’ouverture et de fermeture des services, visites aux malades : raconter des expériences vécues)
    - Modalité d’accès au traitement : Pouvez-vous nous parler des modalités d’accès au traitement ? (gratuité, paiement de certains services etc.)
    - Changements dans votre vie : quels sont les changements (positifs, négatifs etc.) de la COVID-19 dans votre vie ?
    - Suggestions pour une meilleure prise en charge : quelles sont les suggestions que vous pouvez-vous faire pour une meilleure prise en charge des cas de la COVID-19 dans les structures de soins ?

3. **Vécu après hospitalisation**

    - Mode de sortie de l’hôpital : libération par l’administration de l’hôpital, fuite et raisons de la fuite (inefficacité du traitement, incapacité de faire face au coût, etc.)
    - Appréciation de son état de santé : guéri et en bonne santé, guéri avec séquelles, description des symptômes liés à la maladie COVID-19.

4. **Autres choses à ajouter : avez-vous autres choses à ajouter ?**
